# Nicotinic Acetylcholine Receptor Involvement in Inflammatory Bowel Disease and Interactions with Gut Microbiota

**DOI:** 10.3390/ijerph18031189

**Published:** 2021-01-29

**Authors:** Lola Rueda Ruzafa, José Luis Cedillo, Arik J. Hone

**Affiliations:** 1 Laboratory of Neuroscience, Biomedical Research Center (CINBIO), University of Vigo, 36310 Vigo, Spain; lolarrzg@gmail.com; 2 Department of Pharmacology and Therapeutics, Universidad Autónoma de Madrid, 28034 Madrid, Spain; jose_cedillo_mireles@hotmail.com; 3 MIRECC, George E. Whalen Veterans Affairs Medical Center, Salt Lake City, UT 84148, USA; 4 School of Biological Sciences, University of Utah, Salt Lake City, UT 84112, USA

**Keywords:** nicotinic acetylcholine receptors, α7 and α9 nicotinic receptor subtypes, cholinergic anti-inflammatory pathway, gut-brain axis, gut microbiome, dysbiosis, inflammatory bowel disease, COVID-19

## Abstract

The gut-brain axis describes a complex interplay between the central nervous system and organs of the gastrointestinal tract. Sensory neurons of dorsal root and nodose ganglia, neurons of the autonomic nervous system, and immune cells collect and relay information about the status of the gut to the brain. A critical component in this bi-directional communication system is the vagus nerve which is essential for coordinating the immune system’s response to the activities of commensal bacteria in the gut and to pathogenic strains and their toxins. Local control of gut function is provided by networks of neurons in the enteric nervous system also called the ‘gut-brain’. One element common to all of these gut-brain systems is the expression of nicotinic acetylcholine receptors. These ligand-gated ion channels serve myriad roles in the gut-brain axis including mediating fast synaptic transmission between autonomic pre- and postganglionic neurons, modulation of neurotransmitter release from peripheral sensory and enteric neurons, and modulation of cytokine release from immune cells. Here we review the role of nicotinic receptors in the gut-brain axis with a focus on the interplay of these receptors with the gut microbiome and their involvement in dysregulation of gut function and inflammatory bowel diseases.

## 1. Nicotinic Acetylcholine Receptors

### 1.1. Nicotinic Acetylcholine Receptors, Composition, Subtypes, and Pharmacological Properties

Nicotinic acetylcholine receptors (nAChRs) are ligand-gated ion channels ubiquitously expressed throughout the central (CNS) and peripheral (PNS) nervous systems [1,2]. Nicotinic receptors are composed of five individual subunits that assemble in pentameric fashion to form a central ion-conducting channel [3,4]. There are 17 individual subunits, designated by Greek letters, and include α1–α10, β1–β4, δ, ε, and γ. Because of the number and diversity of subunits, numerous distinct nAChR subtypes are possible but can nevertheless be classified into two broad categories: heteromeric subtypes composed of α and β subunits and homomeric subtypes composed of α subunits only. Most heteromeric subtypes contain α and β subunits, for example α3β4, a subtype highly expressed by ganglionic neurons of the PNS [5]. However, heteromeric nAChRs composed strictly of α subunits have also been described and include α9α10 [6,7,8] and α7α8 subtypes [9]. Adding to the diversity of potential subtypes, more than one α or β subunit may be present in a given nAChR complex such as α3β2β4* receptors (the asterisk denotes the potential or known presence of additional subunits in native receptor complexes) which are expressed by rodent adrenal chromaffin cells [10] and neurons of superior cervical and nodose ganglia [11,12]. Homomeric receptor subtypes include α7, α8, α9, and α10 [13,14,15]. It should be noted that α8 subunits are not expressed in mammals and homomeric α10 nAChRs have only been reported in nonmammalian organisms [16].

Each of the various nAChR subtypes possesses different pharmacological and biophysical properties including sensitivities to the neurotransmitters acetylcholine and choline, desensitization properties, and permeabilities to cations [17,18,19,20]. Receptor subtypes that contain the β2 subunit such as α3β2, α4β2, and α6β2 have generally been found to be more sensitive to activation by acetylcholine than the closely related α3β4, α4β4, and α6β4 subtypes [21,22]. Subtypes that contain the β2 subunit are insensitive to the acetylcholine precursor and metabolite choline whereas those containing β4 subunits are weakly activated by choline [23]. By contrast, choline is a full agonist of homomeric α7 nAChRs [24] and a partial agonist of α9 and α9α10 subtypes [8,13,25]. Nicotinic receptors are so named because they are activated by the tobacco plant alkaloid nicotine, but curiously, α9 and α9α10 nAChRs are not activated by nicotine and instead are inhibited by this ligand [8,13,25].

### 1.2. Nicotinic Acetylcholine Receptor Expression by Sensory and Autonomic Ganglion Neurons that Innervate the Gut

Innervation of the gut by neurons of the inferior ganglion of the vagus nerve (nodose ganglion) and dorsal root ganglion (DRG) neurons provide the CNS with sensory information concerning the physiological state of the gut. Although the functional characterization of the nAChRs expressed by nodose ganglion neurons using subtype-selective ligands is lacking, immunoprecipitation assays suggest the presence of several subtypes that contain α2, α3, α4, α5, β2, or β4 subunits [12]. Pharmacological and electrophysiological assays of lumbar DRG neurons from rat suggest that these neurons mainly express α3β4*, α6β4*, and α7 nAChRs [26,27,28]. Innervation of mouse gut by DRG neurons is provided by ganglia located at levels T8-L1 and L6-S1 [29] and have been shown to express α4β2*, α7, and α3β4* nAChRs based on receptor sensitivities to subtype-selective antagonists [30,31]. The functional role of nAChRs in DRG neurons is poorly understood, but α3β4*, α6β4*, and α7 nAChRs have been reported to be expressed by putative nociceptors and may therefore be involved in nociception [28,32,33]. Additionally, α7 nAChRs located on DRG neuron terminals in the dorsal horn of the spinal cord modulate the release of glutamate and have been proposed to be involved in nicotine mediated analgesia [34].

The main nAChR subtypes expressed by autonomic nervous system (ANS) neurons almost certainly contain the α3 subunit as evidenced by CHRNA3 gene knockout mice that show perinatal mortality and severe ANS dysfunction [35,36]. However, sparse functional information is available concerning the exact subtypes expressed by both ANS and enteric nervous system (ENS) neurons innervating the gut. Immunohistochemical studies of ENS plexuses in mice, rats, and guinea pigs suggest neuronal expression of a heterogenous population of nAChRs that contain α3, α5, β2, β4, or α7 subunits [37,38,39]. Functional assays of mouse myenteric plexus neurons demonstrated the presence of at least α3β2* and α3β4* but transcripts for α7 nAChRs were also present [40]. Similar results were found for neurons of the submucosal plexus in guinea pig [41]. Lastly, immunohistochemical studies of myenteric plexuses of mouse colon revealed the expression of α3 subunits in glial cells that also express nitric oxide synthase II [42]. Stimulation of glial cells with the nicotinic agonist dimethylpiperazine increased the production of nitric oxide which functions as a signaling molecule between glia and myenteric neurons. Glial cells and neurons thus coordinate regulation of ion transport in the epithelia through stimulation of nAChRs and the production of nitric oxide. Table 1 lists the expression patterns of nAChRs in neurons that innervate gut structures.

Alterations in the expression patterns of α3β4 nAChRs in neurons of the ANS can result is dysregulation of gut function in humans. Several neurological conditions such as idiopathic, paraneoplastic, and diabetic autonomic neuropathies are associated with the presence of receptor binding (blocking) autoantibodies in patient serum [54]. In autoimmune ganglionopathies where autoantibodies against the α3 subunit are produced, gross ANS dysfunction occurs [55]. Similarly, patients with megacystis microcolon intestinal hypoperistalsis syndrome show significantly decreased expression of the α3 subunit [56], and patients with diverticular disease show decreased β4 subunit mRNA expression in the myenteric plexus [53]. These studies indicate an essential role of α3-containing nAChRs in the gut-brain axis.

## 2. The Gut-Brain Axis

### 2.1. Neural Communication between the Brain and the Gut

**Figure 1:**
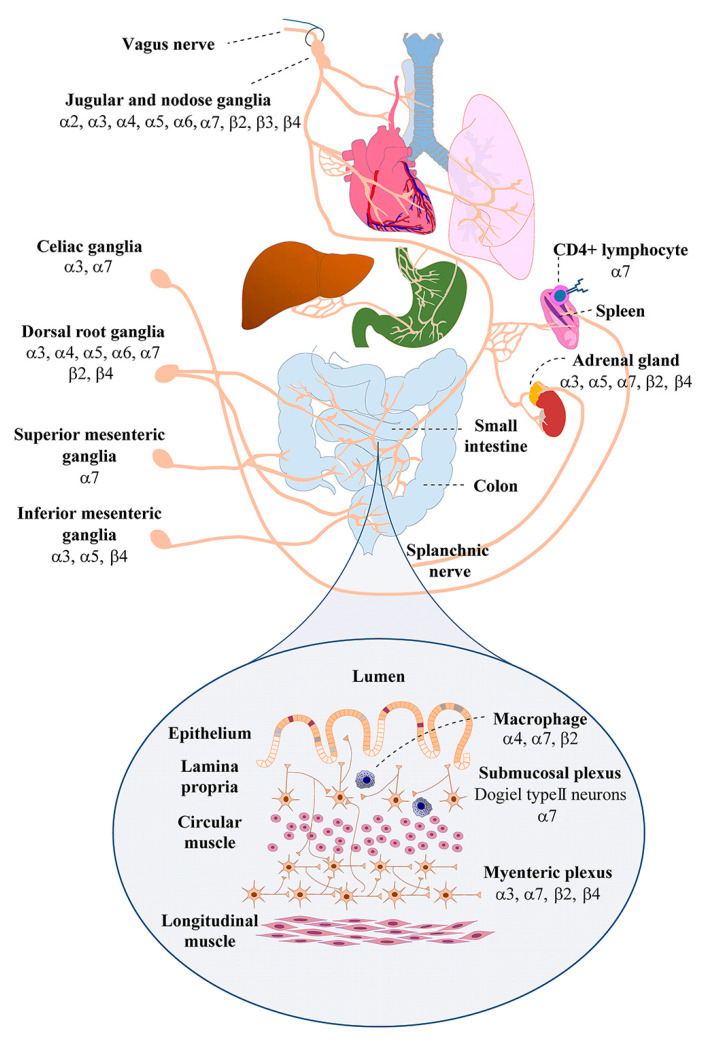
Cartoon representation depicting organs and structures of the gastrointestinal tract and the neurons that innervate them that express nAChR subunits. The inset in the lower part of the cartoon details the structures of the intestines; the myenteric and submucosal plexuses are shown along with select cell types.

It has been well established that a bi-directional relationship exists between the CNS and the gut, and influences myriad pathological conditions from psychiatric to gastrointestinal disorders [57,58]. This ‘gut-brain axis’ controls a number of physiological processes via the brain, autonomic, and enteric nervous systems. Some of the principal components of this system are the vagus nerve, the hypothalamic-pituitary-adrenal axis (HPA axis), and the immune and circulatory systems. Critical to the bi-directional communication between the brain and the gut are neurons that innervate gut structures and the neurotransmitters they release for communication and autocrine/paracrine functions. Neurons of dorsal root and nodose ganglia along with intrinsic primary afferent neurons (Dogiel Type II neurons) of the ENS provide sensory functions to gut structures and relay information concerning gut homeostasis to the CNS [43,57]. Ganglionic neurons of the ANS found in the superior and inferior mesenteric ganglia, celiac, middle and inferior cervical ganglia provide direct PNS innervation to visceral organs although those that specifically innervate structures of the gut are largely found in the celiac, superior and inferior mesenteric ganglia [59] (Figure 1). Direct, local control of gut function is mediated almost entirely by the ENS or the gut-brain which is made up of neural networks or plexuses and include the submucosal and myenteric plexuses [59]. Each of these gut-brain systems is involved in maintaining gut homeostasis and responding to alterations in gut function including those that cause gastrointestinal inflammation.

### 2.2. Inflammatory Control in the Gut Involves the Vagus Nerve and α7 nAChRs

The cholinergic anti-inflammatory pathway (CAP) is referred to as the neuroinflammatory reflex in which the nervous and immune systems ‘cooperate’ to control excessive inflammation, and one mechanism by which this occurs is through activity of the vagus nerve. The vagus nerve is composed of 80% sensory afferent fibers and 20% motor efferent fibers [60]. Vagal nerve fibers innervate the gastrointestinal tract, lungs, heart, pancreas, adrenal glands, and liver and are responsible for the control/modulation of heart rate, digestion, intestinal movement, hormone and neurotransmitter secretion. Correct function of this nerve is essential for numerous physiological processes of the gut-brain axis [61]. Activation of vagal efferents leads to the release of acetylcholine in visceral organs with the exception of the spleen as this organ is innervated by the splenic nerve, which is adregenergic [62]. The splenic nerve releases noradrenaline and activates adrenergic receptors expressed by a specific subpopulation of resident CD4^+^ T-cells that are capable of synthesizing and releasing acetylcholine that, in turn, activates resident macrophage expressed α7 nAChRs to inhibit the release of pro-inflammatory cytokines [63]. The anti-inflammatory effects of α7 nAChR activation have been observed through stimulation of enteric macrophages through vagal nerve activity [63,64,65]. This anti-inflammatory mechanism occurs via activation of α7 nAChRs, recruitment and activation of Janus kinase-2 (JAK-2), and subsequent phosphorylation of signal transducer and activator of transcription-3 (STAT-3) which dimerizes and translocates to the nucleus to inhibit pro-inflammatory cytokine gene expression including tumor necrosis factor-α (TNF-α) and interleukin-6 (IL-6) among others [66,67]. Additionally, activation of α7 nAChRs is associated with inhibition of Nf-κB nuclear translocation [68,69] and activation of the phosphoinositide 3-kinase/protein kinase B (PI3K/Akt) signaling pathways [70,71]. Inhibition of these pathways disrupts signaling through the inflammasome complex [72] and ultimately results in the suppression of TNF-α, Il-6, IL-1β and other pro-inflammatory cytokine secretion. At the systems level, vagal-nerve stimulation has been shown to reduce plasmatic TNF-α levels after lipopolysaccharide (LPS) injection in mice and α7 nAChRs were demonstrated to be a key player in this anti-inflammatory effect [73,74].

Several studies have shown that stimulation of α7 by acetylcholine, choline, nicotine, other agonists and positive allosteric modulators (PAMs) reduces the production of pro-inflammatory cytokines and improves outcomes in animal models of endotoxemic shock [74,75,76,77,78]. The anti-inflammatory role of this receptor is further supported by studies utilizing specific antagonists of α7 receptors, CHRNA7 knock-out mice [63,74,79,80], or overexpression of its dominant-negative duplicated form dupα7 [81] which has only been found in humans. Elevated expression levels of dupα7 in human large and small intestines are associated with inflammatory bowel disease (IBD) [82]. Control of inflammation through the CAP has been demonstrated in animal models of human disease including sepsis, IBD, arthritis, hemorrhagic shock, asthma, and pancreatitis [75,83,84,85,86,87,88,89]. In humans, the importance of the role α7 nAChRs play in the CAP and the regulation of exacerbated inflammation has been shown in sterile endotoxemia [90,91] and sepsis [92]. Activation of the CAP via vagal-nerve stimulation is currently used to treat depression [93], epilepsy [94], stroke [95], and migraines [96]. Vagal-nerve stimulation may also be potentially useful in treating Crohn’s disease, ulcerative colitis, and other inflammatory bowel conditions [97] as has been demonstrated in rodent models of irritable bowel syndrome (IBS) [98] and postoperative ileus [99].

Inflammatory bowel disease is a highly prevalent and multifactorial disorder characterized by chronic inflammation of the gastrointestinal tract and significantly affects the quality of life of patients who suffer from it. The two main types are ulcerative colitis, which is limited to the colon, and Crohn’s disease which can affect any section of the intestinal tract [100]. The vagus nerve plays a role in regulating intestinal inflammation in IBD [101], and the proposed mechanism involves ENS neurons and macrophages located in the submucosal plexus [102]. Release of acetylcholine by the vagus nerve contacting ENS neurons decreases the release of TNF-α, IL-1β, IL-6, and IL-18 by submucosal macrophages expressing α7 nAChRs. In dysbiosis and pathologies such as ulcerative colitis, lymphocytes and macrophages are recruited to the site of inflammation where adhesion molecules are over expressed [103]. In a mouse model of colitis, nicotine suppressed the expression of mucosal addressin cell-adhesion molecule-1 (MAdCAM-1) protein in the mucosal venules of the inflamed colon [104]. In the mouse dextran sodium sulfate (DSS) model of colitis, nicotine reduced lumbar DRG neuron hyperexcitability through activation of α7 nAChRs [105]. Electrical stimulation of the vagal nerve in a mouse model of endotoxemia reversed LPS-induced decreases in tight-junction proteins, via an α7-mediated mechanism, and increased intestinal permeability [106]. Furthermore, intraperitoneal injection of nicotine reduced gut permeability by maintaining localization of intestinal tight-junction proteins after burn-induced gut injury in mice [107]. These finding have led to consideration of a potential protective role of nicotine on bowel wall integrity. However, nicotine also induces significant increases in triglycerides, LDL-cholesterol, and serum glucose along with a decrease in HDL-cholesterol in animals fed a high-fat diet and increased plasmatic levels of certain cytokines raising concerns about its usefulness as an anti-inflammatory therapeutic in IBD [108]. However, other subtype-selective agonists of nAChRs have also shown beneficial effects in animal models of IBD.

Treatment with galantamine, a PAM of nAChRs, succeeded in preventing ulcers and reducing inflammatory mediators such as intracellular adhesion molecule-1 (ICAM-1) in the 2,4,6-trinitrobenzene sulfonic acid (TNBS) model of colitis in rats [109]. The effects of galantamine were abolished by the α7 nAChR antagonist methyllycaconitine. Similarly, use of the α7-selective agonist PNU-282987 improved oxidative enzyme myeloperoxidase activity and reduced IL-6 and IFN-γ levels in the mouse DSS model of colitis [110]. Subsequent treatment with methyllycaconitine reversed the beneficial effects of PNU-282987. Varenicline, a non-selective agonist of α7 nAChRs, improved colonic motility and the cholinergic response in a rat IBS model [111]. Other α7-selective agonists including encenicline and AR-R17779 have shown anti-inflammatory effects in mouse models of colitis and postoperative ileus. Encenicline reduced the infiltration of immune cells into inflamed colonic tissue in TNBS- and DSS- induced colitis [112], and AR-R17779 stimulated the CAP and reduced NF-κB transcription in peritoneal macrophages in postoperative ileus [99]. These studies indicate an important role of α7 nAChRs in IBD. Nevertheless, other studies have reported that stimulation of α7 nAChRs did not reduce intestinal inflammation although the hyperalgesia associated with colonic inflammation was reduced [113]. Overall, however, selective stimulation of α7 nAChRs has shown to be effective in reducing signs and symptoms of disease in a variety of bowel conditions characterized by excessive inflammation. Table 2 lists the effects of activation of α7 nAChRs on IBD. Other nAChR subtypes including α4β2* have been reported to be expressed by a subset of intestinal and peritoneal macrophages that do not express α7 receptors and are not directly involved in the anti-inflammatory effects of the CAP but instead serve a phagocytotic function in the gut [114].

### 2.3. Nicotinic Acetylcholine Receptor Subunits α9 and α10 are Novel Players in IBD

Although the role of the α7 nAChR in IBD has been well studied and firmly established, recently nAChRs containing α9 and α10 subunits have emerged as new targets for treating inflammation. It has been shown that inhibiting the α9α10 receptor with the selective antagonist α-conotoxin RgIA reduced the severity of inflammation in the DSS model of colitis in mice [118]. RgIA and its analogs have been shown to have disease-modifying effects in a number of neuropathic and inflammatory disease models including sciatic nerve injury, diabetic neuropathy, and neuropathies associated with the use of the anti-cancer drugs paclitaxel and oxaliplatin [119,120,121,122]. An important mechanism through which RgIA exerts the observed therapeutic effects is by inhibiting the recruitment of lymphocytes and macrophages to damaged nerve tissues, although the exact mechanisms by which this occurs are currently unknown. However, experiments with nicotine, acetylcholine, or choline in a human monocyte cell line (U937) and mouse peripheral blood mononuclear cells showed that ATP-mediated release of IL-1β, through nAChRs containing α7, α9 or α10 subunits, is inhibited by these nicotinic ligands [123,124]. Phosphocholine, a molecule structurally similar to choline, also inhibited ATP-evoked currents and IL-1β release in U937 cells through α7 and α9α10 nAChRs [123,125,126].

## 3. Bacterial Types in the Gastrointestinal Tract

### 3.1. The Gut Microbiome Plays an Important Role in Communication between the Nervous System and the Gut

A critical component of the gut-brain axis is the make-up of the microbiota found in the different compartments of the gastrointestinal tract. Among the different strains of bacteria present are *Lactobacillus* and *Streptococcus,* found in the stomach and duodenum, *Lactobacillus, Streptococcus, Bacteroides*, *Bifidobacterium,* and *Fusobacteria* in the jejunum and ileum, and *Bacteroides*, *Bifidobacterium, Streptococcus, Eubacteria*, *Clostridium, Vellionella, Ruminococcus, Pseudomonas,* and *Lactobacillus,* among others, in the colon [127]. These bacteria have multiple roles including protective, structural, and metabolic functions for example through the fermentation of dietary fiber into short-chain fatty acids (SCFAs) and the synthesis of B and K vitamins [128,129]. Short-chain fatty acids play an important role in regulating inflammation in the intestines through inhibition of the NF-κB pathway and reduction of macrophage-produced pro-inflammatory cytokines [130,131]. Alterations in the levels of these commensal bacteria can result in intestinal dysbiosis (an imbalance in the populations of intestinal microbiota). Table 3 lists some of the commensal bacteria found in the lower gastrointestinal tract and their roles.

### 3.2. Gut Dysbiosis

Gastrointestinal dysbiosis is associated with a number of pathophysiological conditions including neurodegenerative diseases, psychiatric conditions, diabetes, obesity, autism, and IBD [146]. Alterations in the normal populations of intestinal microbiota can allow the proliferation of harmful bacterial strains and the toxins they produce. Celiac disease and IBS are associated with a decrease in intestinal microbial diversity in general, with alterations in *Firmicutes/Bacteroidetes* ratio and in members of the *Proteobacteria* phylum [147,148]. For instance, elevated levels of endotoxins in the bloodstream such as LPS, derived from the outer membrane of gram-negative bacteria, is a common alteration that can cause a severe immune system response that leads to systemic inflammation and sepsis. Peripheral blood mononuclear cells from patients with IBS show elevated levels of pro-inflammatory cytokine release when challenged with LPS from *Escherichia coli* [149]. Similarly, *Clostridium difficile*, the bacteria responsible for diarrhea associated with overuse of certain antibiotics and the etiology of pseudomembranous colitis, attacks the lining of the intestine through the release of toxins A and B. Both toxins induce damage to the intestinal epithelium, increase permeability of the mucosal barrier, and generate an inflammatory response [150,151].

### 3.3. Effects of nAChR Stimulation by Nicotine on Intestinal Microbiota Populations

As mentioned above, the composition of commensal intestinal microbiota is essential for proper gastrointestinal function. Alterations in the proportion of certain bacterial strains produce negative impacts that lead to the onset, progression, and/or maintenance of IBDs. In relation to nAChRs, results from several studies have shown a disruptive effect from nicotine on the composition of intestinal microbiota populations in mice [108,152]. During a 9-week smoking cessation period, an increase in *Firmicutes* and *Actinobacteria* and a decrease in *Bacteroidetes* and *Proteobacteria* was found in human fecal samples [153]. In mice, chronic oral administration of nicotine increased bacterial alpha-diversity including members of the *Lactobacillus* and *Lachnospiraceae* genera and *Firmicutes* phylum [108]. Interestingly, administration of nicotine in the drinking water of mice showed a sex-dependent effect on the bacterial composition of the intestinal microbiome [152]. The relative abundance of bacteria from the *Christensenellaceae* and *Anaeroplasmataceae* families showed significant reductions in female mice after a 13-week exposure to nicotine whereas males showed decreased *Dehalobacteriaceae* bacteria. Similarly, daily exposure to tobacco smoke increased cecal *Clostridium clostridiforme* and decreased *Lactoccoci*, *Ruminococcus albus*, *Enterobacteriaceae* and *Bifidobacterium* compared to controls in mice and rats [154,155]. In addition, SCFAs such as butyrate, propionate, and acetate were reduced by the effect of smoke exposure [154]. Activation of the free fatty acid receptor 3 (FFA3) by SCFAs has been shown to reduce colonic motility and abolish chloride secretion involving nAChRs via G protein-coupled receptors in rats [156,157]. Thus, the composition of gut microbiota is essential for maintaining the ability of the host organism to regulate intestinal inflammation and respond to pathogenic organisms that target the intestinal tract. Table 4 lists the effects of nicotine on the bacterial composition of gut microbiota.

## 4. Potential Involvement of nAChRs in COVID-19 and Associated Dysbiosis

### The Pathophysiology of COVID-19 May Involve α7 nAChRs and Inhibition of the CAP

In late December of 2019, a novel strain of coronavirus was reported in Hubei province, China in patients with viral pneumonia and was determined to be similar to other coronaviruses that causes severe acute respiratory syndrome (SARS) [158]. The sequence of this virus, SARS-CoV-2, was quickly determined and showed high similarity to other members of the coronavirus family including SARS-CoV-1 and RaTG13 but with one notable difference [158]. Unlike SARS-CoV-1 and RaTG13, SARS-CoV-2 contains additional residues (681-PRRA-684) between the S1 and S2 domains of the spike protein [159,160]. These residues serve as a cleavage site for the furin enzyme and have been proposed to impart increased infectiousness of SARS-CoV-2 relative to other members of the SARS-CoV family. This hypothesis is controversial, however, and requires further investigation [161,162].

**Figure 2:**
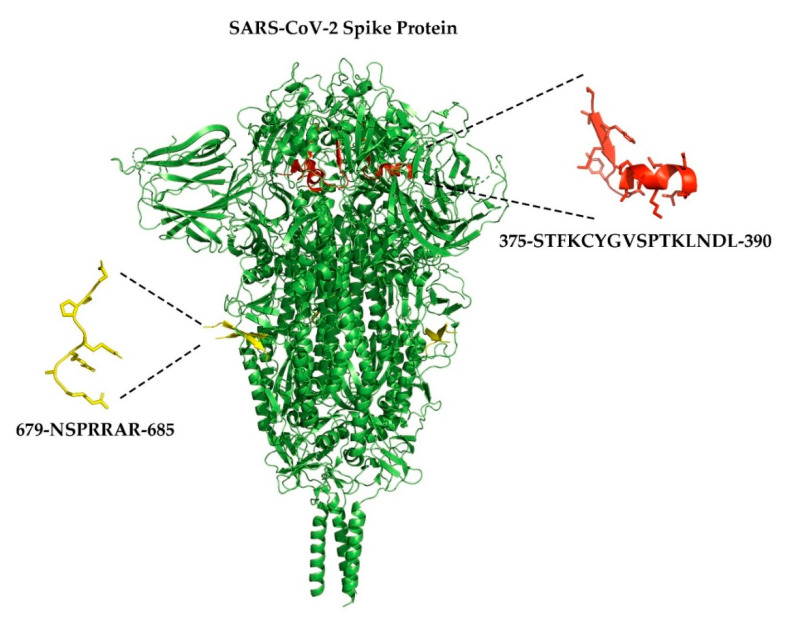
Cartoon representation of the SARS-CoV-2 spike protein trimer (green) showing the proposed domains that interact with α7 and α9α10 nAChRs. Note that residues 675-QTNSPRRARSVA-686 are unresolved in this structure. Residues highlighted in yellow are those that show homology with sequences of the three-finger neurotoxins from *Elapidea* serpents including α-bungarotoxin from *Bungurus multicintus* and α-cobratoxin from *Naja naja* species [163]. Residues highlighted in red have also been proposed to interact with α7 and α9α10 nAChRs [164]. Rendition of the spike protein was accomplished using PyMOL [166] and adapted from Cai et al., 2020 (PDB:6XR8) [167]; rendition of the NSPRRAR sequence was adapted from Daly et al., 2020 (PDB: 7JJC) [168].

Researchers at the Pasteur Institute and the Sorbonne in Paris, France observed that the sequence of the furin cleavage site along with seven residues (674-YQTQTNS-680) upstream and one arginine-685 residue downstream were similar to a motif found in neurotoxins from *Elapidea* serpents [163] (Figure 2). This motif allows serpent neurotoxins to bind to and inhibit nAChRs, most notably α7 nAChRs, which led Changeux and his colleagues to hypothesize that inhibition of α7 receptors by the SARS-CoV-2 spike protein may contribute to the pathophysiology of COVID-19 and specifically to elevated levels of cytokines. Computational modeling experiments later suggested that the spike protein may potentially interact with receptors that contain α7 subunits and/or α9-containing subtypes [164]. Given the possibility that the spike protein interacts with α7 nAChRs, inhibition of this receptor has been proposed as a contributor to the so-called ‘cytokine storm’ through inhibition the CAP [163,164,165].

SARS-CoV-2 not only produces acute respiratory distress but has shown a propensity for inducing severe dysfunction of neurological, pulmonary, cardiovascular, and gastrointestinal systems. Some patients develop acute gastrointestinal distress including diarrhea and vomiting which initially led to the assumption that patients with IBD would experience more severe gastrointestinal symptoms than those without due to the presence of significant angiotensin-converting enzyme-2 receptor expression in the ileum and colon as suggested by analysis of transcriptomics data [169]. In addition, immunosuppressive therapies are often first-line treatments for IBD. However, analysis of clinical data has, in fact, suggested the contrary leading to speculation that immunotherapies with biologics and other immune system modulators may actually reduce COVID-19-related symptoms by suppressing the cytokine storm [170,171,172,173]. Similarly, pharmacological stimulation of α7 receptors and the CAP has been proposed as a mechanism to ‘calm the storm’ [174]. As discussed above, α7 is highly involved in inflammatory conditions of the gastrointestinal tract and low expression levels of α7 receptors are associated with worse outcomes in Crohn’s, other IBDs, and sepsis [82,92]. The systemic presence of an antagonist of α7 receptors would almost certainly worsen the gastrointestinal symptoms associated with COVID-19 by inhibiting the anti-inflammatory actions of the CAP. Therefore, treatment with an agonist such as nicotine might be beneficial and do two things: (1) bind to the ligand-binding site of α7 receptors and compete with or inhibit spike protein binding while simultaneously activating the receptor, and (2) stimulate the CAP to inhibit the cytokine storm. Indeed, such a treatment has been proposed by several authors [165,175,176].

The gastrointestinal symptoms associated with COVID-19, as experienced by some patients, including increased prevalence of diarrhea and vomiting may cause alterations in the gut microbiome and influence the severity of the disease [177,178]. COVID-19 has been shown to be associated with reduced bacterial diversity in the gut and increased prevalence of harmful strains of bacteria [179]. Analysis of fecal samples from patients with COVID-19 found differences in the gut microbiome in those with high fecal levels of SARS-CoV-2 mRNA compared to those with low levels of mRNA [180]. Specifically, patients with high levels of viral mRNA showed increased prevalence, relative to those with low or no fecal viral mRNA, of *Collinsella aerofaciens* and *Morganella morganii*, bacteria that are associated with opportunistic infections in humans. By contrast, patients with low (or no) detectable levels of viral mRNA showed higher levels of bacteria known to produce SCFAs including members of *Parabacteroides*, *Bacteroides*, and *Lachnospiraceae* families. Therefore, alterations in the gut microbiome in patients with COVID-19 may influence the course and severity of the disease. Treatment of COVID-19 with probiotics to combat such alterations has been suggested as a way to ameliorate COVID-19 symptoms [178,181].

## 5. Conclusions

The aim of this Review was to evaluate the involvement of nAChRs in the gut-brain axis by examining their role in different physiological and pathophysiological processes of the gastrointestinal tract. The extensive expression of nAChRs by neurons that innervate gastrointestinal organs influences numerous physiological processes including gut motility, sensory detection of signaling molecules released by other neurons, immune cells, and bacteria. Importantly, control of gut inflammation through α7 and α9 nAChRs, the vagus nerve, and the CAP is essential. We note that there is a surprising lack on information concerning several important areas of nAChR research on the gut-brain axis. First, sparse information is available detailing the functional nAChR subtypes expressed by ENS neurons and glial cells. Research on the role of glial cells in general in gut function is also lacking. Determining the subtype composition of these receptors is important in the context of designing pharmacotherapeutics that treat IBD. Gene knock-out of CHRNA3 in mice produces gross ANS dysfunction, and certain human diseases of the gut involve production of antibodies against α3 and β4 subunits. It is highly likely that ENS neurons express the α3β4* subtype, but it is possible that multiple different subtypes containing α3 and β4 subunits are present, for example α3β2β4*, α3β4α5* and α3β2*, which are highly expressed by other PNS neurons. Each of these nAChRs subtypes may show different sensitivities to ligands. Nevertheless, it is critical that potential drugs used to treat IBD be devoid of activity on α3β4 subtypes to avoid secondary side effects associated with excessive activation or inhibition of α3β4 receptors. Additionally, sparse information is available concerning the expression of the α7 subtype in the ENS and essentially nothing is known about the expression of subtypes containing α9 subunits. Secondly, information about the interaction of commensal bacteria and enterotoxins, produced by pathogenic strains, with nAChRs is lacking. It is known that bacteria from *Firmicutes* species and from *Bacteroides* and *Eubacteria* genera and other commensal bacteria produce SCFAs. These fatty-acid molecules reach millimolar concentrations in the intestines and are involved in the esterification of choline. Choline and its various derivatives have been shown to modulate the release of cytokines from murine macrophages and human monocytes through α7- and/or α9-containing nAChRs as well as decrease chloride secretion from intestinal epithelia. Alterations in SCFA-producing populations of bacteria may therefore affect activities of nAChRs expressed by sensory neurons innervating the gut, ENS neurons, and immune cells and ultimately affect regulation of gut homeostasis. Lastly, although numerous studies detailing the effects of nicotine on gut microbiota have been reported, little information is available concerning the effects of other nAChR compounds including those listed in Table 2. In the context of pharmacotherapy of IBD with nAChR compounds, it is important to determine the potential effects these compounds might have on commensal bacterial populations. Clearly, more research is needed to elucidate the nAChR subtypes expressed in the gut-brain axis, their interactions with bacteria, and the effects of experimental nicotinic IBD therapeutics on commensal bacteria.

## Figures and Tables

**Table 1:** nAChR expression in neurons that innervate the gastrointestinal tract

Neural Structure	nAChR Subunits ^a^	Functional nAChRs ^b^	Target Organ in the Gastrointestinal Tract	Ref.
Nodose ganglia ^c^	α2, α3, α4, α5, α6, α7, β2, β3, β4	α3β4 *	Proximal small intestine and colon	[12,43,44]
Dorsal root ganglia ^c,e^	α3, α4, α5, α6, α7, β2, β4	α3β4 *, α4β2 *, α6β4 *, α7	Small and large intestines	[26,27,43,45,46]
Celiac ganglia ^c^	α3, α7	α3 *^,f^, α7 ^f^	Distal esophagus, stomach, proximal duodenum, liver, biliary system, spleen, adrenal glands	[47,48]
Superior mesenteric ganglia ^c^	α7	α7 ^f^	Duodenum, jejunum, ileum, cecum, appendix, ascending colon, proximal transverse colon	[47]
Inferior mesenteric ganglia ^c,d^	α3, α5, β4	α3β4 *	Distal transverse, descending, and sigmoid, colon, rectum, upper anal canal	[49]
Inferior hypogastric plexus ^c,d^	α3, β4, α7	unknown	Urogential organs, pelvic viscera	[50,51]
Myenteric plexus ^c,d,e^	α3, α5, α7, β2, β4	α3β2 *, α3β4 *, α7	Circular and longitudinal muscles of the gut wall, submucosa, epithelia, stomach, small and large intestines, colon	[37,39,52,53]

^a^ Subunits detected by molecular biology techniques; ^b^ nAChR subtypes detected by functional assays; ^c^ rodent; ^d^ guinea pig; ^e^ human; ^f^ probable functional expression; * denotes the potential presence of other subunits.

**Table 2:** Effects of nAChR ligands on murine models and human patients with IBD

Ligand	Mechanism of Action	Disease-modifying Mechanisms	Effects on IBD	Ref.
Nicotine	Non-selective agonist	Suppression of MAdCAM-1; reduced regenerative spike action-potentials	Decreased signs and symptoms of DSS-induced colitis in mice Reduced colonic DRG neuron hyperexcitability in DSS-induced colitis in mice	[30,104]
Galantamine	Non-selective PAM	Reduced NF-κB, TNF-α levels, MPO, and neutrophil infiltration	Decreased signs and symptoms of TNBS-induced colitis in mice	[109]
PNU-282987	α7-selective agonist	Reduced infiltration of leucocytes Reduced infiltration of macrophages, and reduced levels of IL-6, and IFN-γ	Attenuated colonic inflammation in DSS-treated mice Decreased signs and symptoms of DSS-induced colitis in mice	[110,115]
PNU-120596	α7-selective PAM	Decreased IL-1β and TNF-α in LPS-treated mice	Decreased symptoms related to anxiety and depression in mice	[116]
GTS-21	partial α7 agonist	Decreased TNF-α in plasma	Probable decreased colonic inflammation in patients with ulcerative colitis	[117]
AR-R17779	α7 agonist	Reduced colonic infiltration of CD4^+^ and CD8^+^ lymphocytes; inhibition of macrophage activation	Decreased signs and symptoms of TNBS-induced colitis in mice Decreased signs and symptoms of postoperative ileus in mice	[99,114]
Encenicline	partial α7 agonist	Reduced colonic infiltration of macrophages, neutrophils, and B lymphocytes	Decreased signs and symptoms of TNBS- and DSS induced colitis in mice	[112]
RgIA	α9 antagonist	Reduced levels of colonic TNF-α	Decreased signs and symptoms of DSS-induced colitis in mice	[118]

Dextran sodium sulfate, DSS; a 2,4,6-trinitrobenzene sulfonic acid, TNBS; oxidative enzyme myeloperoxidase, MPO; mucosal vascular addressin cell adhesion molecule-1, MAdCAM-1.

**Table 3:** Bacterial types, location within the gastrointestinal tract, and function

Bacteria	Location in the GI Tract	Functional Role	Ref.
*Lactobacillus*	Stomach, duodenum, jejunum, ileum, colon	Improved digestion and absorption of nutrients; inhibition of the growth of pathogens by activating the immune system ^a,b^	[132,133,134]
*Streptococcus*	Jejunum, ileum, colon	Modulation of the immune system through altered cytokine release from immune cells^c^	[135,136]
*Bacteroides*	Jejunum, ileum, colon	Production of SCFAs involved in energy homeostasis and regulation of intestinal inflammation^d^	[137]
*Bifidobacterium*	Jejunum, ileum, colon	Inhibition of the growth of pathogens by activating the immune system; amino-acid and vitamin synthesis^d^	[138,139]
*Veillonella*	Colon	Production of SCFAs involved in energy homeostasis^c,d^	[140]
*Eubacteria*	Colon	Production of SCFAs involved in energy homeostasis and regulation of intestinal inflammation^c^	[141]
*Clostridia*	Colon	Participation in resistance to the colonization of pathogens; production of SCFAs^c^; maintenance of gut homeostasis^c^	[142,143,144]
*Peptostreptococcus*	Colon	Maintenance of epithelial barrier and modulation of intestinal inflammation^c,d^	[145]

^a^ Dog; ^b^ cat; ^c^ human; ^d^ rodent.

**Table 4:** Effects of nicotine on gut microbiota and their function.

	Effect on Bacterial Levels	Effects on Gut Function	Ref.
Bacteria			
alpha-diversity	Increased in mice	Improvement of gut barrier function by production metabolites and antimicrobial substances	[108]
*Lactobacillus*	Increased in mice	Improvement of gut barrier function and prevent inflammation by production of SCFAs, lactate and antimicrobial substances	[108]
*Lachnospiraceae*	Increased in mice	Improvement of gut barrier function by production of beneficial metabolites such as SCFAs	[108]
*Christensenellaceae*	Decreased in female mice	Development of metabolic syndrome	[152]
*Anaeroplasmataceae*	Decreased in female mice	Alteration of the intestinal transit	[152]
*Dehalobacteriaceae*	Decreased in male mice	Development of metabolic syndrome	[152]

## Data Availability

Published data for the x-ray crystallography structures of the SARS-CoV-2 spike protein and its peptide fragment are archived in the Protein Data Bank (PDB:6XR8 and PDB:7JJC) at rcsb.org.
